# Reference Genes for qPCR Analysis in Resin-Tapped Adult Slash Pine As a Tool to Address the Molecular Basis of Commercial Resinosis

**DOI:** 10.3389/fpls.2016.00849

**Published:** 2016-06-16

**Authors:** Júlio C. de Lima, Fernanda de Costa, Thanise N. Füller, Kelly C. da Silva Rodrigues-Corrêa, Magnus R. Kerber, Mariano S. Lima, Janette P. Fett, Arthur G. Fett-Neto

**Affiliations:** ^1^Plant Physiology Laboratory, Center for Biotechnology and Department of Botany, Federal University of Rio Grande do Sul Porto Alegre, Brazil; ^2^Biological Sciences Department, Regional Integrated University of Alto Uruguai and Missões (URI-FW) Frederico Westphalen, Brazil

**Keywords:** resin, *Pinus*, gene expression, normalizer genes, terpene synthase

## Abstract

Pine oleoresin is a major source of terpenes, consisting of turpentine (mono- and sesquiterpenes) and rosin (diterpenes) fractions. Higher oleoresin yields are of economic interest, since oleoresin derivatives make up a valuable source of materials for chemical industries. Oleoresin can be extracted from living trees, often by the bark streak method, in which bark removal is done periodically, followed by application of stimulant paste containing sulfuric acid and other chemicals on the freshly wounded exposed surface. To better understand the molecular basis of chemically-stimulated and wound induced oleoresin production, we evaluated the stability of 11 putative reference genes for the purpose of normalization in studying *Pinus elliottii* gene expression during oleoresinosis. Samples for RNA extraction were collected from field-grown adult trees under tapping operations using stimulant pastes with different compositions and at various time points after paste application. Statistical methods established by *geNorm, NormFinder*, and *BestKeeper* softwares were consistent in pointing as adequate reference genes *HISTO3* and *UBI*. To confirm expression stability of the candidate reference genes, expression profiles of putative *P. elliottii* orthologs of resin biosynthesis-related genes encoding *Pinus contorta* β-pinene synthase [*PcTPS-(*−*)*β*-pin1*], *P. contorta* levopimaradiene/abietadiene synthase (*PcLAS1*), *Pinus taeda* α-pinene synthase [*PtTPS-(*+*)*α*pin*], and *P. taeda* α-farnesene synthase (*Pt*α*FS*) were examined following stimulant paste application. Increased oleoresin yields observed in stimulated treatments using phytohormone-based pastes were consistent with higher expression of pinene synthases. Overall, the expression of all genes examined matched the expected profiles of oleoresin-related transcript changes reported for previously examined conifers.

## Introduction

Pine oleoresin is a major source of terpenes, consisting of turpentine (mono- and sesquiterpenes) and rosin (diterpenes) fractions. Oleoresin represents a key element of defense in conifers and its production can be affected by biotic and abiotic factors, which include mechanical injury, pathogen attack, water availability, seasonality, and chemical stimulating treatments (Rodrigues-Corrêa and Fett-Neto, 2012). Higher oleoresin yields are economically desirable, since oleoresin derivatives make up a valuable source of materials for chemical industries (Rodrigues-Corrêa et al., 2012). Currently, oleoresin is extracted for commercial purposes from living trees, often by the bark streak method, in which bark removal is done periodically, followed by application of chemical stimulant paste containing sulfuric acid and other chemicals on the freshly wounded exposed surface (Rodrigues-Corrêa et al., 2013). Chemical stimulant pastes may contain phytohormones or their precursors (e.g., ethylene, auxin, salicylic acid), metal ions (enzyme activators or co-factors, such as potassium and iron), or reactive oxygen species generators (e.g., paraquat), all of which increase resin production and/or facilitate its flow by activating defense responses (Rodrigues-Corrêa and Fett-Neto, 2012).

Terpenes of conifers are mostly biosynthesized by secretory tissue in the inner surface of resin ducts present in conifer bark and xylem, in the cambium zone, and associated vascular tissues (Back, 2002). Resin accumulates within the ducts, exerting positive pressure on its walls, thereby being exuded upon wounding (Trapp and Croteau, 2001). Terpenes are derived from isopentenyl pyrophosphate (IPP), produced either through the mevalonate pathway in the cytosol-endoplasmic reticulum or by deoxi-xylulose-5-phosphate pathway in the plastids (Phillips and Croteau, 1999).

The pathway of terpene biosynthesis may be divided in various stages. First, IPP is synthesized and isomerized to dimethyl allyl diphosphate (DMPP). Then, prenyltransferases catalyze the condensation of these two C5-units to geranyl diphosphate (GPP), forming monoterpenes (C_10_), and the subsequent 1′-4 head-to-tail additions of isopentenyl diphosphate generate farnesyl (FPP) and geranylgeranyl (GGPP) diphosphate, which fuse to originate sesquiterpenes (C_15_) and diterpenes (C_20_), respectively. Next, the prenyl diphosphates undergo a range of cyclizations driven by terpene synthases (TPS) and other modifying reactions to produce the immense diversity of different terpenoid metabolites (Rodrigues-Corrêa et al., 2013).

Although chemical stimulant pastes have been intensely studied in *Pinus elliottii* Engelm var. *elliottii* (slash pine) aiming at higher yields of oleoresin (Rodrigues et al., 2008, 2011; Rodrigues and Fett-Neto, 2009; Rodrigues-Corrêa and Fett-Neto, 2013), knowledge of genes and their regulatory pattern for terpene biosynthesis in commercial forests are still unknown. Acquiring this information would be a crucial aspect to understand the biochemical and physiological basis of oleoresin production. Hence, the aim of this study was to evaluate the stability of 11 putative reference genes for the purpose of normalization in studying *P. elliottii* gene expression during oleoresinosis under field conditions, undergoing tapping operation with different stimulant pastes and at different time points after paste application. Statistical methods established by *geNorm* (Vandesompele et al., 2002), *NormFinder* (Andersen et al., 2004), and *BestKeeper* (Pfaffl et al., 2004) were comparatively used to analyze the data. In addition, to confirm stability of the candidate reference genes, expression profiles of putative *P. elliottii* orthologs of conifer genes encoding several terpene synthases involved in biosynthesis of different resin components were monitored following stimulant paste application.

## Materials and methods

### Plant material and resin tapping process

Approximately 16 years-old slash pine (*P. elliottii* Engelm. var. *elliottii*) trees with a diameter at breast height ranging from 65 to 90 cm, grown at Irani Celulose forest installations, Balneário Pinhal, RS, Brazil (30°14′S and 50°14′W), were used in the experiments. Resin tapping was performed according to a protocol previously established (Rodrigues et al., 2011) based on Pio and Valente (1998), using the “bark streak” system (Stubbs et al., 1984). Each stimulant paste contained 20% of sulfuric acid in aqueous solution and rice husk powder as an inert substrate to optimize paste consistency. Single modifications in the composition of the resin stimulant paste were done by including 1.0 mM of the synthetic auxin 1-naphthaleneacetic acid (Treatment name: NAA), 500 mM potassium sulfate (Treatment name: POTASSIUM) or 3% of 2-chloroethylphosphonic acid (Treatment name: CEPA) (v/v of active ingredient, an ethylene releasing agent). A negative control without paste application (bark streak only) was also included. The treatments and rationale for application are listed in Table 1.

**Table 1:** **Oleoresin stimulant paste adjuvant treatments tested under field conditions on bark wounds of *P. elliottii***.

**Treatment**	**Adjuvant**	**Mode of action**
POTASSIUM	Potassium sulfate	Potassium is a cofactor of conifer terpenoid synthases (Savage et al., 1994; Trapp and Croteau, 2001; Rodrigues et al., 2011)
NAA	Naphthaleneacetic acid	Promotes ethylene biosynthesis (increases ACC synthase gene transcription) and may induce resin duct formation, as a result of cambial activity stimulation (Chae and Kieber, 2005; Song et al., 2005; Rodrigues and Fett-Neto, 2009)
CEPA	2-Chloroethylphosphonic acid	Precursor of ethylene, signaling molecule involved in stress-induced responses, increases biosynthesis of mono- and diterpenes (reviewed in Rodrigues-Corrêa et al., 2013)

**Figure 1:**
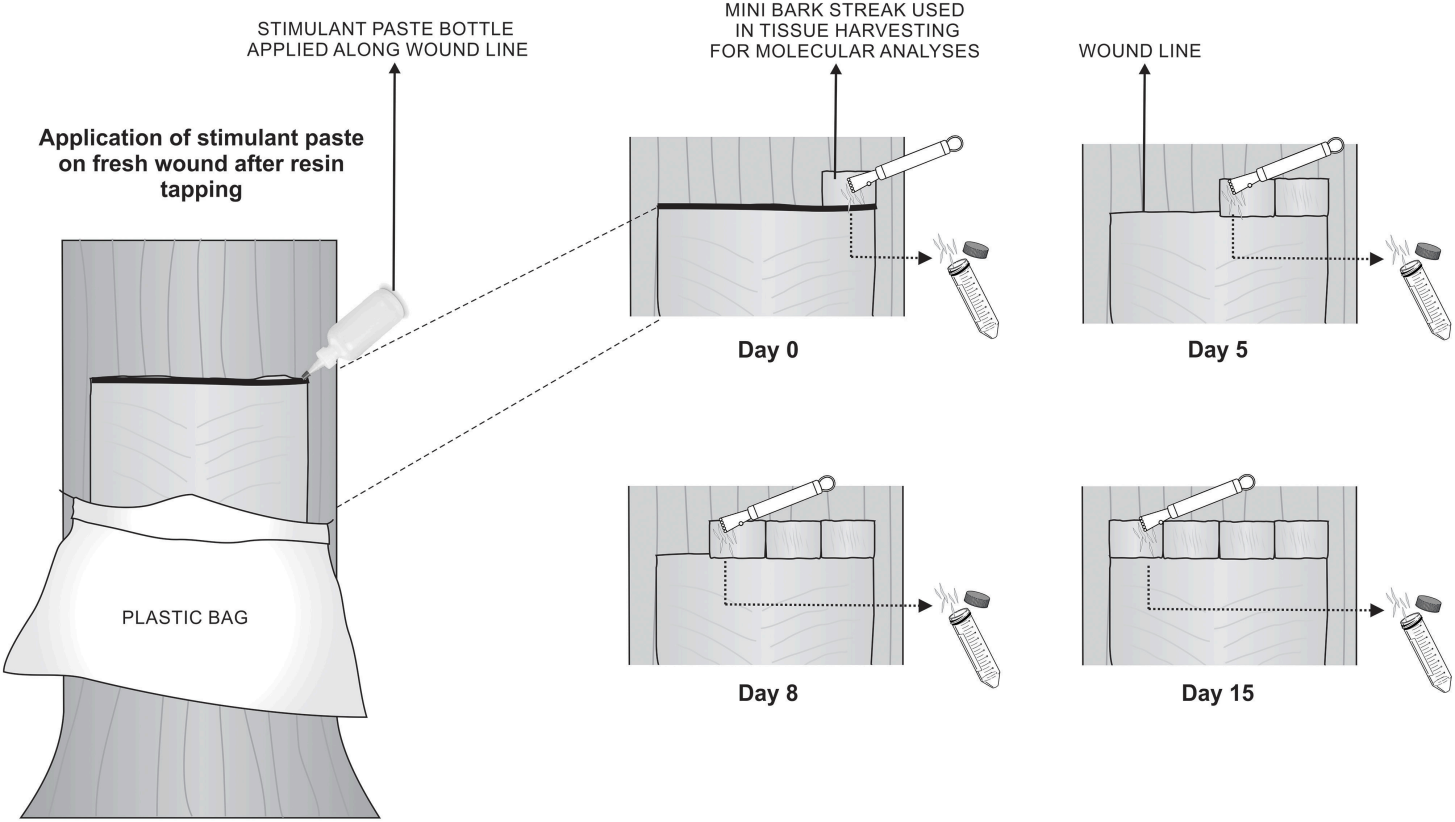
**Schematic representation of RNA extraction from vascular cambium of slash pine trees**. Process of tapping and application of the respective stimulant paste was performed on day 0. On days 0, 5, 8, and 15 after tapping, a small novel bark streak was made right above the previous one, phloem tissues were removed using a wood chisel and the cambium was exposed. RNA was extracted from vascular cambium region and immediately frozen in liquid nitrogen for subsequent analysis. Stimulant paste treatments were: NAA, POTASSIUM, CEPA, or control (bark streak only).

Each treatment consisted of five trees randomly selected, each located in the inner portion of the forest, excluding the trees in forest borders. The trees used in this study had been previously tapped for resin production biweekly for one year with the respective stimulant paste. For this experiment, the process of tapping and application of stimulant paste directly on the fresh wounds occurred only once. Immediately after paste application and after 5, 8, and 15 days, a novel mini bark streak was made right above the previous one, phloem tissues were removed using a wood chisel and the cambium was exposed (Figure 1). Vascular cambium was scrapped and the removed tissue was immediately frozen in liquid nitrogen and kept at −80°C until RNA extraction. Tissue had to be harvested slightly above the wound streak due to poor quality of RNA obtained in the wound line itself. The mini bark streak approach was also chosen because it is known that stimulant chemicals in paste can move upward and sideways in the tree, affecting surrounding tissues (Brown and Nix, 1975; Rodrigues-Corrêa and Fett-Neto, 2012).

Biomass of resin yielded by each treatment was estimated as an average for one bark streak based on the winter season production of 80 individual trees of the same stand.

### Isolation of RNA and first strand cDNA synthesis

Total RNA was isolated from vascular cambium of slash pine plants using Concert plant RNA reagent (Life Technologies), following manufacturer indications. Total RNA (500 ng) was treated with 1U DNAse I and DNAse I reaction buffer (Life Technologies) before cDNA synthesis. Total RNA concentration was determined using an UV spectrophotometer (Nanodrop, Thermo Scientific) and nucleic acid quality was checked with 2100 Bioanalyzer instrument (Agilent). For the amplification of the candidate reference genes, all treated RNA was reverse transcribed using oligo-dT primers and M-MLV reverse transcriptase (Life Technologies) in a final volume of 20 μl. The final cDNA products were diluted 50-fold in RNAse-free distilled water prior to use in qPCR.

### PCR primer design

Eleven genes were selected as candidate internal controls and are described in Table 2. Genes were previously reported as reference genes in embryogenesis studies with other conifer species such as *P. taeda, P. pinaster*, and *Picea abies* (Gonçalves et al., 2005; de Vega-Bartol et al., 2013). Primers for *ACT, ATUB, CYP* and *eEF* genes were designed with Primer3 v 0.40 (Applied Biosystems, Foster City, CA, USA) using the default parameters of the software. Primers for all other candidate genes were employed as reported in conifer embryogenesis studies (Gonçalves et al., 2005; de Vega-Bartol et al., 2013). All primer pairs were checked for their probability to form dimers and secondary structures. Expression profiles of putative *P. elliottii* orthologs of resin biosynthesis-related genes were also examined following different stimulant paste application. To that end, the best pair of reference genes selected in this study was used as normalizer and the data were analyzed by the different softwares. Primers for amplifying transcripts of the various terpene synthase genes are listed in Table S1.

**Table 2:** **Description of candidate reference genes, primers, and amplicons for internal control gene selection in *P. elliottii***.

**Gene**	**Function**	**Primer sequence (5′-3′) (forward/reverse)**	**Amplicon length (bp)**	**Amplification efficiency**	**GenBank accession number or Reference**
*ACT* (actin)	Structural constituent of cytoskeleton, ATP binding	CACTGCACTTGCTCCCAGTA/GGTCTTGGCAATCCACATCT	130	1.961	AY172979 (*Pt*)
*AK* (adenosine kinase)	Phosphotransferase activity, cysteine biosynthesis	ATCTTTAGGGCTATTTCTTC/TTACT GAGGCATTGATGA	127	1.845	de Vega-Bartol et al., 2013
*ATUB* (α-tubulin)	Structural constituent of cytoskeleton, microtubule-based processes	CCGTTTCTTCGCAGTTTTTC/CAAGCATTTCCAACCTGGAT	119	1.878	KF158848 (*Pt*)
*CYP* (cyclophilin)	Protein folding and trafficking, pre-mRNA splicing, extracellular signaling molecules	TGCAAAGACAGAGTGGTTGG/GCAATAACTACGGGCTTGGA	129	1.870	EF532602 (*Pt*)
*eEF (*elongation factor)	Translational elongation	ACAACCACTGGGCATTTGAT/ACGTTCAGCCTTGAGCTTGT	138	1.866	FN256567 (*Pp*)
*eIF4AII* (translation initiation factor 4AII)	Mediates the recruitment of ribosomes to mRNA, delivery of an RNA helicase to the 5′ region of the mRNA	ATTCAGGTGGGTGTTTTCTCT/GTGTGATTGCCAGGGTCTC	202	1.943	Gonçalves et al., 2005
*HEATS* (homologous to a heat shock protein)	Response to heat and high light, protein folding	GGCAGAATACAAGAATGG/TTGAAGTTCCGTCAGTTA	120	1.944	de Vega-Bartol et al., 2013
*HISTO3 (*histone 3)	Cell proliferation, DNA Binding, RNA methylation	GCTGAGGCTTACCTTGTG/CCAGTTGTATATCCTTAGGCATAA	94	1.877	de Vega-Bartol et al., 2013
*REDUC* (homologous to a ether reductase protein)	Ether reductase protein, response to cadmium	AGGAAGACATTGGAACATT/CAGAGTATTGGCAGGAAG	92	1.878	de Vega-Bartol et al., 2013
*SAND* (SAND protein)	Intracellular vesicular transport, biogenesis, and vacuole signaling	GATCGTGGAGCAATTTGG/TTATCGACATATCATCCTCATT	86	1.832	de Vega-Bartol et al., 2013
*UBI* (ubiquitin)	Signaling complexes for protein degradation, translation control, DNA repair, endocytosis regulation, protein traffic	GATTTATTTCATTGGCAGGC/AGGATCATCAGGATTTGGGT	149	1.870	de Vega-Bartol et al., 2013

*Pt, P. taeda; Pp, P. pinaster*.

### Amplification and sequencing of the reference genes

All genes were amplified from the pooled cDNA and reactions were carried out in total volumes of 50 μl, containing template cDNA (1:50 dilution in water), 10 X PCR Buffer, 10 mM dNTPs, 50 mM MgCl_2_, 10 μM each primer (forward and reverse) and 5 U/μl Platinum Taq polymerase. The cycling conditions for the amplification of PCR products involved an initial denaturation of 95°C/5 min, followed by 40 cycles of 95°C/30 s, primer-specific annealing temperature/30 s and 72°C/30 s.

Specificity of each primer pair was verified by sequencing amplicons in an ABI 3100 automatic DNA sequencer (Life Technologies). Proper sizes of amplification products were further confirmed by gel electrophoresis on 1% agarose gel and visualization after staining with ethidium bromide.

### qPCR

The synthesized cDNA was diluted 1:50 with RNAse-free distilled water, and 10 μl of the diluted cDNA was used as a template for quantitative real-time PCR analysis. PCR reactions were carried out in a technical quadruplicate for each sample and performed in a total volume of 20 μl as described previously (de Almeida et al., 2010). All analyses were performed using three biological replicates for each time point and individual treatment. Reactions were performed in fast optical 48-well reaction plates 0.1 ml (MicroAmp™–Applied Biosystems) using a StepOne™ Real-Time PCR System (Applied Biosystems) according to the manufacturer instructions. PCR amplifications included an initial denaturation step of 5 min at 95°C, followed by 40 cycles of 15 s at 95°C, 10 s at 60°C, and 15 s at 72°C, after which samples were held for 2 min at 40°C for annealing and then heated from 55 to 99°C with a ramp of 0.1°C/s to produce the denaturing curve (or melting curve) of the amplified products. Melting curves were used to validate product specificity. Obtained data were analyzed by the comparative C_q_ (quantitative cycle method) (Livak and Schmittgen, 2001). The PCR efficiency from the exponential phase (Eff) was calculated for each individual amplification plot using the LinReg software (Ramakers et al., 2003). In each plate, the average of PCR efficiency for each amplicon was determined and used in further calculations.

### Selection of potential reference genes

Three softwares, *geNorm* (Vandesompele et al., 2002), *NormFinder* (Andersen et al., 2004), and *BestKeeper* (Pfaffl et al., 2004), were used to select the most stably expressed reference genes during slash pine oleoresinosis. Expression stability of the candidate reference genes was first ranked using *geNorm* and the output was compared to the results of *NormFinder* and *BestKeeper*.

The *geNorm* algorithm calculates the average expression stability (*M*-value) using raw non-normalized expression values, which gives the average pairwise variation (V_n_/V_n+1_) of expression of a particular gene in relation to all candidate reference genes. According to this software, the gene with the lowest *M*-value is considered the most stable. Using an *M*-value below the threshold of 1.5 was initially recommended (Vandesompele et al., 2002), although a maximum value of 0.5 has been proposed for more accurate results whenever possible (Hellemans et al., 2007; Gutierrez et al., 2008). Genes with the lowest *M*-values have the most stable expression. In addition, *geNorm* software calculates the minimal number of reference genes required for normalization based on the geometric mean of their final optimal set. A pairwise variation of 0.15 is accepted as cut-off (Vandesompele et al., 2002), below which the inclusion of an additional control gene is not required for reliable normalization.

*NormFinder* has an algorithm that uses raw non-normalized data in the form of expression values generated using the comparative *C*_q_ method. It enables estimation of the overall variation of the reference normalization genes and the variation between subgroups of the sample set, taking into account intra- and intergroup variations for normalization factor calculations. Genes with lowest values are considered the most stable and are top ranked. Similarly to the *geNorm* method, *NormFinder* reveals a score of expression stability to each gene, which is negatively correlated with the stability of gene expression (Andersen et al., 2004). The best candidate will be the one with the closest to zero inter-group variation, and, at the same time, having the smallest error bars.

*Bestkeeper* uses raw data (*C*_q_-values) and PCR efficiency and ranks the stability of candidate reference genes by performing a statistical analysis of the *C*_q_-values based on three variables: Pearson correlation coefficient (r), standard deviation (SD) and percentage covariance (CV). In this case, any studied gene with the SD higher than 1 is considered inconsistent and should be excluded.

### Validation of reference genes

To further confirm the reliability of the potential reference genes, the relative expression profiles of four genes involved in mono, di and sesquiterpene biosynthesis in pines were examined. Expression profiles of putative *P. elliottii* orthologs (herein referred to as “like-genes”—Table S1) of resin biosynthesis-related genes encoding *P. contorta* β-pinene synthase (*(*−*)*β*pinS1-*like; Hall et al., 2013a), *P. taeda* α-pinene synthase (*(*+*)*α*pinS*-like; Phillips et al., 2003), *P. contorta* levopimaradiene/abietadiene synthase (*LAS1*-like; Hall et al., 2013b), and *P. taeda* α-farnesene synthase (α*FS*-like; Phillips et al., 2003) were examined following stimulant paste application. In these analyses, the normalization of expression data was carried out using the most stable genes identified in this study.

The qPCR amplification conditions were the same as described above. Relative expression levels of the target gene were calculated with the 2^−ΔCq^ method (Livak and Schmittgen, 2001). Values shown represent the average of three biological replicates.

The analyses covered three time points (5, 8, 15 days after wounding) and three different treatments with stimulant pastes (NAA, POTASSIUM, and CEPA), besides the control (bark streak only).

All amplicons of both reference and TPS genes were sequenced to confirm correct identity.

### Statistical analyses

Experiments were conducted in completely randomized layout. Results were analyzed by ANOVA followed by Tukey test or Welch ANOVA followed by Dunnett's C test (between time points within each treatment) or *t*-test (between treatments within each time point), whenever appropriate, using the statistic package SPSS 20.0 for Windows (SPSS Inc., USA). A *P* ≤ 0.05 was used in all cases. Data were expressed as mean ± standard error (S.E.).

## Results

### Resin yield induced by different stimulant paste treatments

Estimated resin biomass exuded per streak was significantly increased in all of the trees treated with resin-stimulant paste compared to the non-stimulated control trees. Although CEPA treated trees produced more resin that those treated with POTASSIUM, overall yields of trees stimulated with the different pastes were very similar (Figure S1).

### Description and expression profile of the candidate reference genes

Total RNA isolated from the 48 samples (including biological replicates) had high quality. Specificity of the primers was confirmed by agarose gel electrophoresis and melting curve analysis (Figure S2). Sequencing of amplicons confirmed the identity of all candidate reference genes (data not shown). Amplification efficiencies of PCRs ranged from 1.832 for *SAND* to 1.961 for *ACT* (Table 2). These numbers provided a good basis for further validation of reference genes.

**Figure 2:**
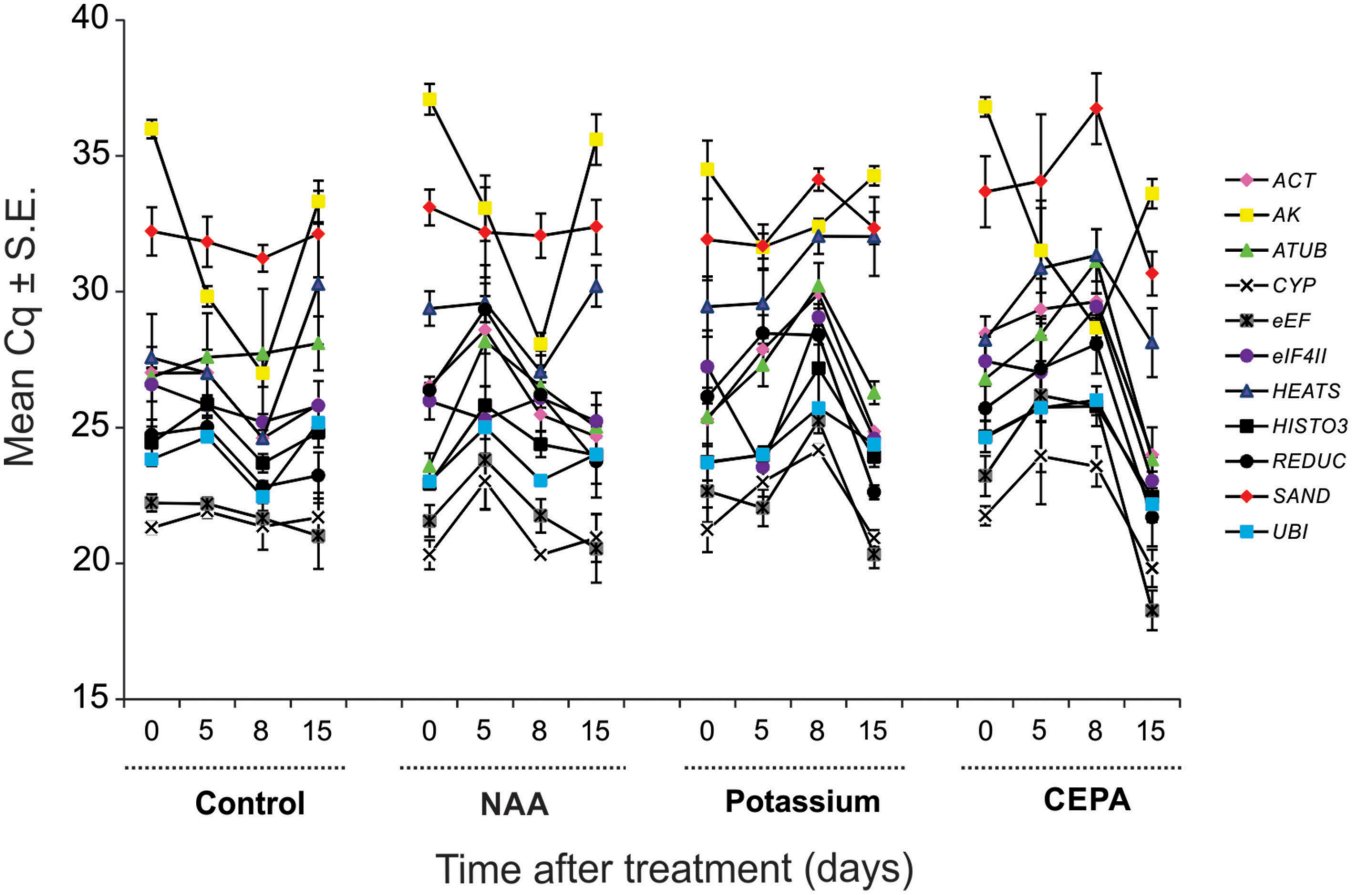
**Expression of tested reference genes along the different treatments and days of analysis presented as *C*q mean value ± standard error (S.E.)**. Legend: pink diamond (*ACT*), yellow square (*AK*), green triangle (*ATUB*), white “X” mark–(*CYP*), gray “X” mark: (*eEF*), purple circle–(*eIF4II*), blue triangle (*HEATS*), dark square: (*HISTO3*), dark circle: (*REDUC*), red diamond: (*SAND*), and blue square: (*UBI*).

The expression level of the genes tested was different. In qPCR, considering all time point values, the reference genes reached mean *C*_*q*_-values (i.e., number of cycles needed for the amplification- related fluorescence to reach threshold level of detection) ranging from 21 to 32, most lying between 22 and 26. *CYP* (mean *C*_q_ ± S.E. = 21.85 ± 0.25) was the most expressed gene and *SAND* (mean *C*_q_ ± S.E. = 32.73 ± 0.33) the least. The expression profiles of all candidate reference genes along the different treatments tested are shown in Figure 2.

### Expression stability of candidate genes

Three different approaches implemented in *geNorm, Normfinder*, and *Bestkeeper* programs were used to determine the expression stability of each candidate gene in comparison to the other candidate genes. Overall, the rankings generated by the three programs were consistent.

#### geNorm

**Figure 3:**
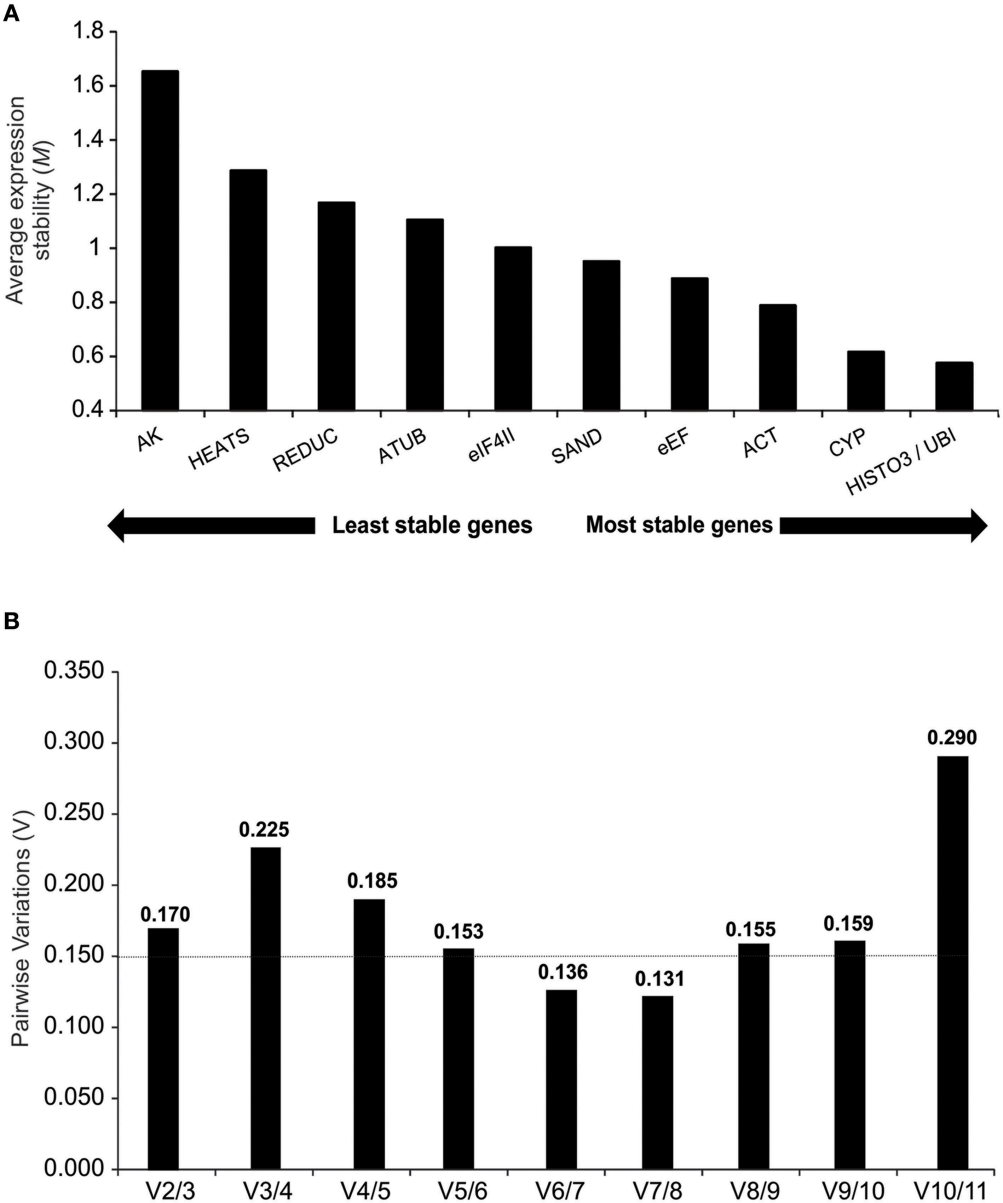
**ge*Norm* descriptive analyses during oleoresinosis in *P. elliottii*. (A)** Average expression stability (*M*) and ranking of the 11 reference genes. A lower average expression stability *M* value indicates more stable expression. **(B)** Pairwise variation of the candidate genes.The pairwise variation (Vn/Vn+1) was calculated between the normalization factors NFn and NFn+1, with a recommended cut-off threshold of 0.150.

Under different treatment conditions and throughout the sampling period, 10 reference genes showed high expression stabilities with threshold values below 1.5. The exception was the *AK* gene (*M* = 1.65), which was disregarded for further analysis. Among those genes with *M* values below 1.5, *HEATS* (*M* = 1.28) was the least stable gene and *HISTO3* and *UBI* (*M* = 0.57) were identified as the best pair of reference genes (Figure 3A).

To generate accurate and reliable results, *geNorm* algorithm finds the optimal number of suitable reference genes required for proper normalization by step wise calculation of the pairwise variation (V_n_/V_n+1_) between two sequential normalization factors, which measures the effect of increasing reference genes required for normalization. Considering the reference value of 0.15 proposed by Vandesompele et al. (Vandesompele et al., 2002), both V6/7 and V7/8 reach it, indicating that six reference genes are required for normalization of gene expression analyses. However, V5/6 showed an acceptable value (0.153), suggesting that five reference genes may also suffice for expression analyses (Figure 3B).

#### NormFinder

Results from this software highlighted *UBI* as the best reference gene (stability value = 0.34), and ranked *CYP* (0.42) and *HISTO3* (0.49) in the second and third positions, respectively, whereas *HEATS* and *AK* were scored as the least stable genes (Table 3). Nevertheless, results showed *UBI* and *HISTO3* at the forefront of the ranking, indicating a good agreement between the results of *NormFinder* and *geNorm* for the data set.

**Table 3:** **Ranking of candidate reference genes in *P. elliottii* in decreasing order of expression stability calculated by *NormFinder* software**.

**Ranking order**	***NormFinder* (stability value ± S.E.)**
1	*UBI* (0.34 ± 0.03)
2	*CYP* (0.42 ± 0.09)
3	*HISTO3* (0.49 ± 0.20)
4	*SAND* (0.50 ± 0.22)
5	*ACT* (0.55 ± 0.10)
6	*eEF* (0.63 ± 0.15)
7	*eIF4II* (0.69 ± 0.17)
8	*ATUB* (0.88 ± 0.39)
9	*REDUC* (0.91 ± 0.50)
10	*HEATS* (0.97 ± 0.57)
11	*AK* (1.93 ± 0.68)

#### BestKeeper

*BestKeeper* was also used to calculate and compare gene expression variation for the candidate reference genes based on their pair-wise correlation with an index value, which is indicated by the Pearson correlation coefficient (*r*). *BestKeeper* calculates the standard deviation (SD) and the coefficient of variation (CV) based on the raw *C*_q_-values of each gene. Because the maximum number of genes analyzed by this algorithm is 10 (Pfaffl et al., 2004), the candidate gene *AK* was excluded from the analyses, since it ranked lower in the previous analyses (*geNorm* and *NormFinder*). The most stable reference genes exhibit the lowest CV and SD and highest *r*. Genes with SD higher than 1 should be considered inconsistent. Only two genes, *UBI* and *HISTO3*, had *SD* ≤ 1 and thus could be considered to be stably expressed. Regarding these two genes, *BestKeeper* recommended *UBI* as the most stable gene with the highest correlation coefficient (0.893) and lowest CV [% *C*_q_] (4.01). The second most stable gene was *HISTO3*, with an *r* of 0.869 and CV of 4.09. The comparison of the eight other candidate reference genes was based on their *r*-value and resulted in a ranking as follows, from the most stable to the least stable: *ACT* > *eEF* > *CYP* > *ATUB* > *SAND* > *REDUC* > *EIF4II* > *HEATS*. The complete ranking is depicted on Table 4. These results were essentially consistent with those obtained using *geNorm* and *NormFinder*.

**Table 4:** ***BestKeeper* descriptive statistical analyses of 10 reference genes of vascular cambium in *P. elliottii* during oleoresinosis, *n* = 48 (total number of samples)**.

**Factors**	**Stability of reference genes**									
	***ACT***	***ATUB***	***CYP***	***eEF***	***eIF4II***	***HEATS***	***HISTO3***	***REDUC***	***SAND***	***UBI***
SD [± Cq]	1.66	1.53	1.10	1.64	1.35	1.53	**0.99**	1.98	1.19	**0.99**
CV [% Cq]	6.20	5.66	5.04	7.28	5.17	5.25	4.09	7.70	3.62	4.01
*r*-value	**0.946**	0.817	0.915	**0.945**	0.787	0.548	0.869	0.793	0.814	0.893
*P*-value	0.001	0.001	0.001	0.001	0.001	0.001	0.001	0.001	0.001	0.001
Ranking	3	6	5	4	9	10	2	8	7	1

*n, number of samples; SD [±Cq], standard deviation of C_q_-values; CV [% Cq], coefficient of variance expressed as percentage of C_q_-values; r, Pearson correlation coefficient; P, probability value. The highest r-values and SDs lower than 1 have been highlighted in bold*.

### Reference gene validation

**Figure 4:**
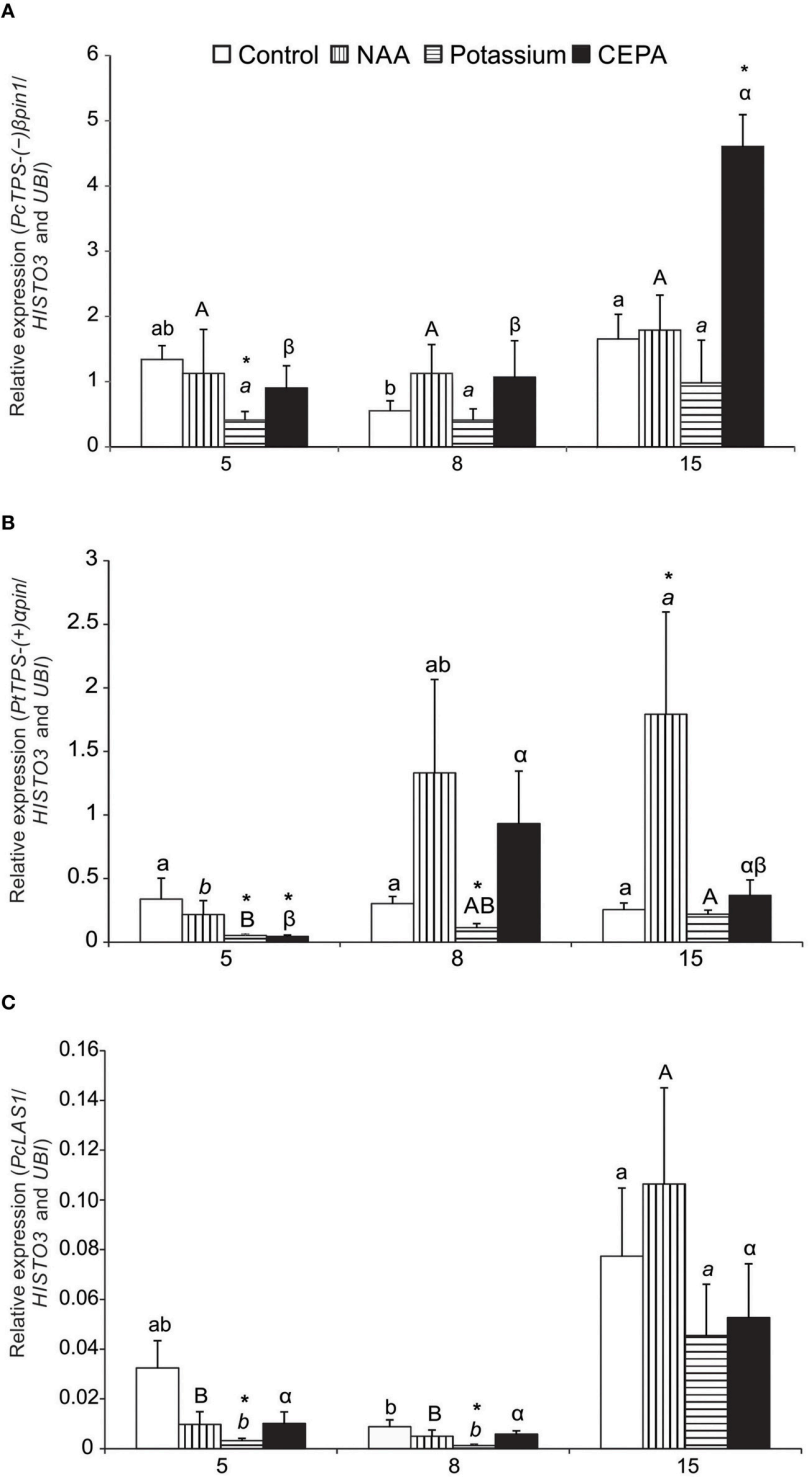
**Relative expression profile of target genes during oleoresinosis in *P. elliottii* 5, 8, and 15 days after treatment**. **(A)**(−)β*pinS1*-like*;* **(B)** (+)α*pinS*-like*;***(C)** *LAS1*-like.The expression profile of the target genes was investigated relative to the best combination of reference genes indicated by *geNorm, NormFinder*, and *BestKeeper* softwares (*HISTO3* and *UBI*). Different lowercase, uppercase, italic, and Greek letters indicate significant difference among the samples of control, NAA, potassium, and CEPA, respectively, by Tukey test or Dunnett's C test (*P* ≤ 0.05). ^*^Indicates that the treatment was significantly different compared to control (bark streak only, with no resin stimulant paste) within the respective time point by *t*-test (*P* ≤ 0.05).

Expression profiles of (−)β*pinS1*-like, *LAS1*-like, (+)α*pinS-*like, and α*FS-*like genes were examined following resin stimulant paste application. The best pair of reference genes identified (*UBI* and *HISTO3*) was used as normalizer. The analyses covered three time points (5, 8, 15 days after treatment) and three different treatments with stimulant paste (NAA, POTASSIUM and, CEPA), besides the control (bark streak only). Sequencing of amplicons confirmed identity of all TPS genes. (−)β*pinS1*-like mRNA expression remained stable after 5 and 8 days, but increased significantly after 15 days in CEPA-treated trees. At this time, expression in CEPA-treated trees was higher than that of the control treatment (Figure 4A). For the other treatments, (−)β*pinS1*-like expression was essentially the same throughout the time course evaluated. Potassium-treated trees had lower expression of (−)β*pinS1*-like compared to control in day 5.

Expression of(+)α*pinS*-like gene also showed increases in expression at later time points, particularly for NAA and CEPA-treated trees (Figure 4B). CEPA-treated trees had a peak in day 8, whereas NAA-treated ones reached a maximum in day 15. At this time, expression of (+)α*pinS*-like in NAA-treated trees was significantly higher than in the corresponding control treatment (Figure 4B). As seen for the (−)β*pinS1*-like gene, expression of *(*+*)*α*pinS*-like in potassium-treated trees was lower compared to the corresponding controls in days 5 and 8.

Expression of *LAS1*-like gene increased over time, peaking at 15 days for all treatments. Significant increases throughout the time course were observed for NAA and potassium-treated plants (Figure 4C). However, there were no differences of treatments within time points in relation to control plants, except for decrease in potassium treatment in days 5 and 8. Compared to the pinene synthase-like genes, *LAS1*-like gene expression was considerably lower, between one and two orders of magnitude (Figure 4).

The α*FS-*like gene expression was only consistently detected in day 15, all treatments being equivalent in level of expression. This gene also showed relatively low expression in days 5 and 8, ranging from 0.05 to 0.06 (data not shown).

## Discussion

The selection of appropriate housekeeping genes is a crucial requisite to successful gene expression profiling based on qPCR. The use of inaccurate reference genes can lead to inconsistent results, particularly when variations in the rate of transcription are small between sample groups (Dheda et al., 2005; Etschmann et al., 2006). Ideal reference gene(s) should exhibit relatively constant levels of RNA steady-state in all tissues/cells or organs and should not be regulated or influenced by the experimental procedures (Radonić et al., 2004; Guenin et al., 2009). Nevertheless, ideal reference genes for all tissues, biological conditions and treatments may be difficult to establish, for the expression of a putative reference gene may vary among the several possible biological situations.

Eleven putative reference genes were selected for expression studies on *P. elliottii* oleoresinosis based on previous reports that indicated them as suitable genes for normalization of expression profiles in different species and in relation to other biological processes (Gonçalves et al., 2005; Wang et al., 2010; de Vega-Bartol et al., 2013). Those 11 candidates were evaluated to find the best set of stable gene normalizers to check gene expression associated with resinosis under the effect of alternative chemical elicitors at different time periods after initial bark wounding. Expression stability was evaluated by three different softwares (*geNorm, NormFinder*, and *BestKeeper*), enabling a more reliable analysis considering the gene expression data. *HISTO3* and *UBI* were identified as the top reference genes using *geNorm* and *BestKeeper*, whereas *NormFinder* ranked *HISTO3* in the third ranking position, ranking *UBI* and *CYP* as the most stable genes. In spite of some minor inconsistencies that are usually observed between these different methods (Gonçalves et al., 2005; Lin and Lai, 2010; Morgante et al., 2011; Chang et al., 2012; Liu et al., 2014), our results were quite consistent regardless of the algorithm employed.

Several studies have shown that the application of more than one reference gene can provide greater accuracy in qPCR experiments with plants (Vandesompele et al., 2002). To determine the optimal number of reference genes required for accurate normalization, the *geNorm* software calculates the pairwise variation (Vn/Vn+1) between the sequential normalization factors. Our results indicated five to six as the ideal number of housekeeping genes, which would increase significantly the experimental costs. However, a number of other studies using this application have resulted in higher pairwise variations (De Ketelaere et al., 2006; Hong et al., 2008; Jian et al., 2008; Fernandez et al., 2011; Han et al., 2012; Kong et al., 2014). The *geNorm* threshold lower than 0.15 is not a strict cut-off but an ideal value to provide guidance in determining the optimal number of reference genes, also requiring an analysis of the trend of changing pairwise variation values (Hong et al., 2008). Depending on the specific conditions of the study, the optimal number of stable controls that should be used for normalization against the target genes has to be modified. In the present study, the experiments were performed in the field, with environmental conditions not subject to control and using individuals derived from seeds, hence with distinct genetic characteristics. Therefore, we suggest that two reference genes (*HISTO3* and *UBI*) are sufficient to normalize samples, since the V2/3 value was relatively close to 0.15 (V2/3 = 0.17) and the stepwise addition of a third reference gene did not result in decrease of the V value closer to 0.15 (V3/4 = 0.22) (Figure 3B).

The high expression stability showed in our experiments for *HISTO3* and *UBI* corroborates data from Tu et al. (2007) in a study with cotton (*Gossypium hirsutum* L.). The expressions of *HISTO 3, UBI 7* and *Gbpolyubiquitin-1* were the most stable during cotton fiber development and somatic embryogenesis. Wang et al. (2013) also selected ubiquitin as one of the most reliable reference gene for gene expression under abiotic stress in cotton, and the same result was also previously seen in other species (Hong et al., 2008; Løvdal and Lillo, 2009; Morgante et al., 2011; Monteiro et al., 2013).

The use of qPCR to examine reference gene expression in conifers is still very limited. To date, studies of normalizing genes in conifers have focused on somatic embryogenesis (Gonçalves et al., 2005; de Vega-Bartol et al., 2013). Expression profiles of four candidate reference genes have been examined in maritime pine (*P. pinaster*) during embryogenesis: *GAPDH, 18S, eIF4AII*, and *UBI* (Gonçalves et al., 2005). However, high variability of the four genes tested ruled out their use as internal standards for qPCR in this developmental process.

De Vega-Bartol et al. (2013) analyzed internal control genes during different steps of somatic embryogenesis in *P. pinaster* and *P. abies* (embryonal mass proliferation, embryo maturation and germination). The best combination for *P. pinaster* included three genes: *EF1, ATUB*, and *HISTO3*. For *P. abies*, four genes were necessary for normalization: *ATUB, AK, CAC*, and *EF1*. However, authors discuss that more accurate results were obtained when each developmental stage was analyzed separately. In spite of the reported developmental stage dependence found in these studies, our results on *P. elliottii* resinosis concerning the use of *HISTO3* as suitable housekeeping gene is in good agreement with *P. pinaster* embryogenesis expression data. This could be an indication that *HISTO3* may represent a useful option to be considered in other conifer studies.

The oleoresin synthesized by *Pinus* is a major source of terpenes, which are biosynthesized by TPS mostly in the cambium zone and associated with vascular tissues. Oleoresin is a well-established defense product in conifers and its biosynthesis may be modulated, at least in part, by wounding and/or chemical stimuli (Rodrigues-Corrêa and Fett-Neto, 2012). Chemical treatments in addition to wounding have been used as an economic strategy explored in resin tapping operations, as a result of the improvement in resin exudation (Rodrigues-Corrêa and Fett-Neto, 2012; Rodrigues-Corrêa et al., 2013).

Various genes encoding TPS have been already isolated and characterized from conifer species (Rodrigues-Corrêa et al., 2013). Four different resin biosynthesis-related genes were evaluated as targets during resin extraction under field conditions for validating the best normalizers identified in the present study (*UBI* and *HISTO3*), as determined by *geNorm, NormFinder* and *BestKeeper* softwares. Expression profiles of *P. elliottii* orthologs of β-pinene (*PcTPS-*(−)β*pin1*), levopimaradiene/abietadiene (*PcLAS1*) synthase, α-pinene synthase (*PtTPS-*(+)α*pin*), and α-farnesene synthase (*Pt*α*FS*) genes were examined following resin stimulant paste application.

Taken together, the main results for expression profiles of the resin biosynthesis-related target genes used in reference gene validation may be summed up as follows: (1) higher expression levels of all genes were observed at later time points; (2) (−)β*pinS1*-like and (+)α*pinS*-like genes were more expressed than those of *LAS1*-like and α*FS*-like genes; (3) significantly higher expression relative to control trees was observed in phytohormone (NAA and CEPA)-stimulated trees, which correlated well with higher resin yields; (4) in spite of its capacity to increase resin yield, potassium-based pastes had almost no stimulatory effect on gene expression.

The kinetics of gene expression showing an increase at later time points is in agreement with the general practice of bark streaking every 15 days, since this would ensure a reactivation of the wound responses that lead to resin production and flow (Rodrigues et al., 2008). *P. elliottii* trees had an increase in monoterpene biosynthesis after 2 weeks of wounding (Popp et al., 1995b). The accumulated biosynthesized resin from the last streak likely flows during the first days after the current streak. In fact, it is commonly observed under field conditions that resin flow is more intense following bark streaking. Presumably, paste stimulants could induce new resin biosynthesis predominantly at transcript level in later time points during the current streak and enzyme activity at earlier periods in the following streak.

The higher expression of (−)β*pinS1* and (+)α*pinS*-like genes compared to *LAS1*-like and α*FS*-like genes within the time frame evaluated is consistent with reported changes in gene expression and enzyme activity for *Abies grandis* after wounding, in which higher gene expression (Northern blot data) and TPS activity were determined for monoterpene synthases (Trapp and Croteau, 2001). In *P. contorta* and *Pinus banksiana* successive bark punches (10 mm diameter) at zero, 2 and 14 days resulted in increased pinene amounts in exuded resin after 2 and 14 days (Clark et al., 2014). Increased monoterpene synthase gene expression was found in *Picea sitchensis* with a peak of expression between 7 and 16 days post-wounding (Byun-McKay et al., 2006). Transcriptomic studies detected that high level of expression of a monoterpene synthase was associated with increased resin production in 27 year-old bark streaked trees of *Pinus massoniana* not treated with stimulant paste (Liu et al., 2015). Although these various patterns of gene expression and terpene production are not from the same species and experimental conditions, they may reflect a highly conserved mechanism of response to damage in conifers. Indeed, well-established chemical stimulants of slash pine resinosis were effective in inducing resin exudation in saplings of a rather primitive gymnosperm that has resin canals only in its bark, *Araucaria angustifolia* (Perotti et al., 2015).

Up-regulation of (−)β*pinS1* and (+)α*pinS*-like was recorded in plants chemically stimulated with CEPA. CEPA is an ethylene-releasing compound and one of the main oleoresin stimulators (McReynolds and Kossuth, 1984). The effect of this compound includes increased biosynthesis of mono and diterpenes in other conifers (Croteau et al., 1987; Popp et al., 1995a,b; Katoh and Croteau, 1998), in good agreement with the results obtained in this study.

Chemical stimulation with NAA also induced increased expression of (+)α*pinS*-like. Auxin can indirectly affect the yield of oleoresin, probably by stimulating the production of ethylene by promoting transcription activation of 1-aminocyclopropane-1-carboxylate synthase (ACS) genes involved in ethylene biosynthesis (Chae and Kieber, 2005) and also by increasing the differentiation of vertical resin ducts (Fahn, 1982). Previous results examining production over a full year showed higher resin yields in plants treated with stimulant paste supplemented with the synthetic auxin 2-4-D (2,4-dichlorophenoxyacetic acid) when compared to control (only bark streak) (Rodrigues and Fett-Neto, 2009). Results of the present study suggest this effect may be at least in part due to increased transcription of a monoterpene synthase gene.

Results with *P. abies* and *P. contorta* showed that potassium takes part in the catalytic mechanism of monoterpene synthases, improving their activity (Savage et al., 1994; Trapp and Croteau, 2001). Previous results with potassium sulfate stimulant paste (500 mM) in *P. elliottii* showed an improvement of pine oleoresin yields when compared to control (Rodrigues et al., 2011). Considering the catalytic role of the metal, which takes place at post-translational level, increased transcript accumulation is not expected with potassium stimulant paste. The limited increases in quantitative gene expression data and the higher yields of resin biomass obtained in the present study with potassium-based paste are in good agreement with this expectation. The lower levels of gene expression observed for potassium paste-treated trees at some time points perhaps could reflect reduced need for transcriptional stimulation as a function of higher enzyme activation.

The absence of major and widespread differences regarding control and stimulated expression in the four target genes examined indicates that probably other levels of regulation of resin biosynthesis are important. These regulatory points may include enzyme activity, protein stability and modification, subcellular compartmentalization, metabolic channeling dynamics, metabolite transport, and substrate availability, all of which are known as being relevant forms of plant secondary metabolism control (Nascimento and Fett-Neto, 2010).

To our knowledge, this is the first systematic analysis for the selection of validated reference genes for qPCR in vascular cambium tissue of field-grown adult individuals of conifers under resin tapping operation, and the first demonstration of increased terpene synthase gene expression during oleoresinosis in slash pine. Our shortlist of reference genes may provide a useful tool to find normalizers for experiments that address other variables and physiological conditions in *Pinus*.

## Author contributions

JD and FD carried out the experiments, analyzed the data, and drafted the manuscript. KR, TF, MK, and ML assisted in different parts of the experimental procedures and sample harvesting. JF revised data analyses and edited the manuscript. AF devised and supervised the study and finalized the manuscript.

## Funding

This work was funded by the National Council for Scientific and Technological Development (CNPq-Brazil), grants 476838/2012-6 and 306079/2013-5.

### Conflict of interest statement

The authors declare that the research was conducted in the absence of any commercial or financial relationships that could be construed as a potential conflict of interest.

## Supplementary material

Table S1 **Description of target genes used to validate the putative reference genes**.

Figure S1 **Resin biomass yield estimated as average per streak**. Data for winter season based on 80 trees per stimulant paste treatment was used to estimate average resin yield per streak (corresponding to a 15 day period). Different letters indicate significant difference between treatments by Tukey test (*P* ≤ 0.05).

Figure S2 **Specificity of qPCR. (A)** Melting curves of the 11 reference genes plus target genes showing a single peak (each representing a technical triplicate). Y-axis: derivative reporter (-Rn) of the melting peak (random units); X-axis: temperature of melting; each vertical line marking the melting peak represents the medium temperature of melting between 80 and 90°C. **(B)** Agarose gel showing PCR amplicons of each gene. Numbers in the gel correspond to the ones in the melting curves. DNA ladder in the gel: 100 bp.

## References

[B1] Andersen C. L. Jensen J. L. Orntoft T. F. (2004). Normalization of real-time quantitative reverse transcription-PCR data: a model-based variance estimation approach to identify genes suited for normalization, applied to bladder and colon cancer data sets. Cancer Res. 64, 5245–5250. 10.1158/0008-5472.can-04-0496 15289330

[B2] Back E. L. (2002). Pattern of parenchyma and canal resin composition in softwoods and hardwoods. J. Wood Sci. 48, 167–170. 10.1007/BF00771362

[B3] Brown C. L. Nix L. E. (1975). Uptake and transport of paraquat in slash pine. Forest Sci. 21, 359–364.

[B4] Byun-McKay A. Godard K. A. Toudefallah M. Martin D. M. Alfaro R. King J.. (2006). Wound-induced terpene synthase gene expression in Sitka spruce that exhibit resistance or susceptibility to attack by the white pine weevil. Plant Physiol. 140, 1009–1021. 10.1104/pp.105.071803 16415217 PMC1400563

[B5] Chae H. S. Kieber J. J. (2005). Eto Brute? Role of ACS turnover in regulating ethylene biosynthesis. Trends Plant Sci. 10, 291–296. 10.1016/j.tplants.2005.04.006 15949763

[B6] Chang E. Shi S. Liu J. Cheng T. Xue L. Yang X.. (2012). Selection of reference genes for quantitative gene expression studies in *Platycladus orientalis* (Cupressaceae) using Real-Time PCR. PLoS ONE 7:e33278. 10.1371/journal.pone.0033278 22479379 PMC3316566

[B7] Clark E. L. Pitt C. Carroll A. L. Lindgren B. S. Huber D. P. W. (2014). Comparison of lodgepole and jackpine resin chemistry: implications for range expansion by the mountain pine beetle, *Dendroctonus ponderosae* (Coleoptera: Curculionidae). PeerJ. 2:e240. 10.7717/peerj.240 24688833 PMC3932820

[B8] Croteau R. Gurkewitz S. Johnson M. A. Fisk H. J. (1987). Biochemistry of oleoresinosis: monoterpene and diterpene biosynthesis in Lodgepole pine saplings infected with *Ceratocystis clavigera* or treated with carbohydrate elicitors. Plant Physiol. 85, 1123–1128. 10.1104/pp.85.4.1123 16665815 PMC1054405

[B9] de Almeida M. R. Ruedell C. M. Ricachenevsky F. K. Sperotto R. A. Pasquali G. Fett-Neto A. G. (2010). Reference gene selection for quantitative reverse transcription-polymerase chain reaction normalization during *in vitro* adventitious rooting in *Eucalyptus globulus* Labill. BMC Mol. Biol. 11:73. 10.1186/1471-2199-11-73 20854682 PMC2955024

[B10] De Ketelaere A. Goossens K. Peelman L. Burvenich C. (2006). Technical Note: validation of internal control genes for gene expression analysis in bovine polymorphonuclear leukocytes. J. Dairy Sci. 89, 4066–4069. 10.3168/jds.S0022-0302(06)72450-X 16960083

[B11] de Vega-Bartol J. J. Santos R. R. Simoes M. Miguel C. M. (2013). Normalizing gene expression by quantitative PCR during somatic embryogenesis in two representative conifer species: *Pinus pinaster* and *Picea abies*. Plant Cell Rep. 32, 715–729. 10.1007/s00299-013-1407-4 23529547

[B12] Dheda K. Huggett J. F. Chang J. S. Kim L. U. Bustin S. A. Johnson M. A.. (2005). The implications of using an inappropriate reference gene for real-time reverse transcription PCR data normalization. Anal. Biochem. 344, 141–143. 10.1016/j.ab.2005.05.022 16054107

[B13] Etschmann B. Wilcken B. Stoevesand K. von der Schulenburg A. Sterner-Kock A. (2006). Selection of reference genes for quantitative real-time PCR analysis in canine mammary tumors using the GeNorm algorithm. Vet. Pathol. 43, 934–942. 10.1354/vp.43-6-934 17099150

[B14] Fahn A. (1982). Plant Anatomy. Oxford: Pergamon Press.

[B15] Fernandez P. Di Rienzo J. Moschen S. Dosio G. A. Aguirrezábal L. N. Hopp H. E.. (2011). Comparison of predictive methods and biological validation for qPCR reference genes in sunflower leaf senescence transcript analysis. Plant Cell Rep. 30, 63–74. 10.1007/s00299-010-0944-3 21076836

[B16] Gonçalves S. Cairney J. Maroco J. Oliveira M. M. Miguel C. (2005). Evaluation of control transcripts in real-time RT-PCR expression analysis during maritime pine embryogenesis. Planta 222, 556–563. 10.1007/s00425-005-1562-0 16034587

[B17] Guenin S. Mauriat M. Pelloux J. Van Wuytswinkel O. Bellini C. Gutierrez L. (2009). Normalization of qRT-PCR data: the necessity of adopting a systematic, experimental conditions-specific, validation of references. J. Exp. Bot. 60, 487–493. 10.1093/jxb/ern305 19264760

[B18] Gutierrez L. Mauriat M. Guénin S. Pelloux J. Lefebvre J.-F. Louvet R.. (2008). The lack of a systematic validation of reference genes: a serious pitfall undervalued in reverse transcription-polymerase chain reaction (RT-PCR) analysis in plants. Plant Biotechnol. J. 6, 609–618. 10.1111/j.1467-7652.2008.00346.x 18433420

[B19] Hall D. E. Yuen M. M. S. Jancsik S. Quesada A. L. Dullat H. K. Li M.. (2013a). Transcriptome resources and functional characterization of monoterpene synthases for two host species of the mountain pine beetle, lodgepole pine (*Pinus contorta*) and jack pine (*Pinus banksiana*). BMC Plant Biol. 13:80. 10.1186/1471-2229-13-80 23679205 PMC3668260

[B20] Hall D. E. Zerbe P. Jancsik S. Quesada A. L. Dullat H. Madilao L. L.. (2013b). Evolution of conifer diterpene synthases: diterpene resin acid biosynthesis in lodgepole pine and jack pine involves monofunctional and bifunctional diterpene synthases. Plant Physiol. 161, 600–616. 10.1104/pp.112.208546 23370714 PMC3561007

[B21] Han X. Lu M. Chen Y. Zhan Z. Cui Q. Wang Y. (2012). Selection of reliable reference genes for gene expression studies using Real-Time PCR in tung tree during seed development. PLoS ONE 7:e43084. 10.1371/journal.pone.0043084 22912794 PMC3422230

[B22] Hellemans J. Mortier G. De Paepe A. Speleman F. Vandesompele J. (2007). qBase relative quantification framework and software for management and automated analysis of real-time quantitative PCR data. Genome Biol. 8:R19. 10.1186/gb-2007-8-2-r19 17291332 PMC1852402

[B23] Hong S.-Y. Seo P. J. Yang M.-S. Xiang F. Park C.-M. (2008). Exploring valid reference genes for gene expression studies in *Brachypodium distachyon* by real-time PCR. BMC Plant Biol. 8:112. 10.1186/1471-2229-8-112 18992143 PMC2588586

[B24] Jian B. Liu B. Bi Y. Hou W. Wu C. Han T. (2008). Validation of internal control for gene expression study in soybean by quantitative real-time PCR. BMC Mol. Biol. 9:59. 10.1186/1471-2199-9-59 18573215 PMC2443375

[B25] Katoh S. Croteau R. (1998). Individual variation in constitutive and induced monoterpene biosynthesis in grand fir. Phytochemistry 47, 577–582. 10.1016/S0031-9422(97)00422-6

[B26] Kong Q. Yuan J. Gao L. Zhao S. Jiang W. Huang Y.. (2014). Identification of suitable reference genes for gene expression normalization in qRT-PCR analysis in watermelon. PLoS ONE 9:e90612. 10.1371/journal.pone.0090612 24587403 PMC3938773

[B27] Lin Y. L. Lai Z. X. (2010). Reference gene selection for qPCR analysis during somatic embryogenesis in longan tree. Plant Sci. 178, 359–365. 10.1016/j.plantsci.2010.02.005

[B28] Liu J. Wang Q. Sun M. Zhu L. Yang M. Zhao Y. (2014). Selection of reference genes for quantitative Real-Time PCR normalization in *Panax ginseng* at different stages of growth and in different organs. PLoS ONE 9:e112177. 10.1371/journal.pone.0112177 25393243 PMC4230945

[B29] Liu Q. Zhou Z. Wei Y. Shen D. Feng Z. Hong S. (2015). Genome-wide identification of differentially expressed genes associated with the high yielding of oleoresin in secondary xylem of Masson pine (*Pinus massoniana* Lamb) by transcriptomic analysis. PLoS ONE. 10:e0132624. 10.1371/journal.pone.0132624 26167875 PMC4500461

[B30] Livak K. J. Schmittgen T. D. (2001). Analysis of relative gene expression data using real-time quantitative PCR and the 2(-Delta Delta C(T)) Method. Methods (San Diego, Calif.) 25, 402–408. 10.1006/meth.2001.1262 11846609

[B31] Løvdal T. Lillo C. (2009). Reference gene selection for quantitative real-time PCR normalization in tomato subjected to nitrogen, cold, and light stress. Anal. Biochem. 387, 238–242. 10.1016/j.ab.2009.01.024 19454243

[B32] McReynolds R. D. Kossuth S. V. (1984). CEPA in sulfuric acid paste increases oleoresin yields. South. J. Appl. For. 8, 168–172.

[B33] Monteiro F. Sebastiana M. Pais M. S. Figueiredo A. (2013). Reference gene selection and validation for the early responses to downy mildew infection in susceptible and resistant *Vitis vinifera* cultivars. PLoS ONE 8:e72998. 10.1371/journal.pone.0072998 24023800 PMC3762845

[B34] Morgante C. V. Guimaraes P. M. Martins A. C. Araujo A. C. Leal-Bertioli S. C. Bertioli D. J.. (2011). Reference genes for quantitative reverse transcription-polymerase chain reaction expression studies in wild and cultivated peanut. BMC Res. Notes 4:339. 10.1186/1756-0500-4-339 21906295 PMC3180468

[B35] Nascimento N. C. Fett-Neto A. G. (2010). Plant secondary metabolism and challenges in modifying its operation: an overview. Meth. Mol. Biol. 643, 1–13. 10.1007/978-1-60761-723-5_1 20552440

[B36] Perotti J. C. Rodrigues-Corrêa K. C. S. Fett-Neto A. G. (2015). Control of resin production in *Araucaria angustifolia*, an ancient South American conifer. Plant Biol. 17, 852–859. 10.1111/plb.12298 25545585

[B37] Pfaffl M. W. Tichopad A. Prgomet C. Neuvians T. P. (2004). Determination of stable housekeeping genes, differentially regulated target genes and sample integrity: BestKeeper – Excel-based tool using pair-wise correlations. Biotechnol. Lett. 26, 509–515. 10.1023/B:BILE.0000019559.84305.47 15127793

[B38] Phillips M. A. Croteau R. B. (1999). Resin-based defenses in conifers. Trends Plant Sci. 4, 184–190. 10322558 10.1016/s1360-1385(99)01401-6

[B39] Phillips M. A. Wildung M. R. Williams D. C. Hyatt D. C. Croteau R. (2003). cDNA isolation, functional expression, and characterization of (+)-alpha-pinene synthase and (-)-alpha-pinene synthase from loblolly pine (*Pinus taeda*): stereocontrol in pinene biosynthesis. Arch. Biochem. Biophys. 411, 267–276. 10.1016/S0003-9861(02)00746-4 12623076

[B40] Pio C. A. Valente A. A. (1998). Atmospheric fluxes and concentrations of monoterpenes in resin-tapped pine forests. Atmos. Environ. 32, 683–691. 10.1016/S1352-2310(97)00327-0

[B41] Popp M. P. Johnson J. D. Lesney M. S. (1995a). Changes in ethylene production and monoterpene concentration in slash pine and loblolly pine following inoculation with bark beetle vectored fungi. Tree Physiol. 15, 807–812. 10.1093/treephys/15.12.807 14965920

[B42] Popp M. P. Johnson J. D. Lesney M. S. (1995b). Characterization of the induced response of slash pine to inoculation with bark beetle vectored fungi. Tree Physiol. 15, 619–623. 10.1093/treephys/15.9.619 14965920

[B43] Radonić A. Thulke S. Mackay I. M. Landt O. Siegert W. Nitsche A. (2004). Guideline to reference gene selection for quantitative real-time PCR. Biochem. Biophys. Res. Commun. 313, 856–862. 10.1016/j.bbrc.2003.11.177 14706621

[B44] Ramakers C. Ruijter J. M. Deprez R. H. Moorman A. F. (2003). Assumption-free analysis of quantitative real-time polymerase chain reaction (PCR) data. Neurosci. Lett. 339, 62–66. 10.1016/S0304-3940(02)01423-4 12618301

[B45] Rodrigues K. C. D. S. Apel M. A. Henriques A. T. Fett-Neto A. G. (2011). Efficient oleoresin biomass production in pines using low cost metal containing stimulant paste. Biomass Bioenerg. 35, 4442–4448. 10.1016/j.biombioe.2011.08.021

[B46] Rodrigues K. C. S. Azevedo P. C. N. Sobreiro L. E. Pelissari P. Fett-Neto A. G. (2008). Oleoresin yield of *Pinus elliottii* plantations in a subtropical climate: effect of tree diameter, wound shape and concentration of active adjuvants in resin stimulating paste. Ind. Crop. Prod. 27, 322–327. 10.1016/j.indcrop.2007.11.010

[B47] Rodrigues K. C. S. Fett-Neto A. G. (2009). Oleoresin yield of *Pinus elliottii* in a subtropical climate: seasonal variation and effect of auxin and salicylic acid-based stimulant paste. Ind. Crop. Prod. 30, 316–320. 10.1016/j.indcrop.2009.06.004

[B48] Rodrigues-Corrêa K. C. D. S. Fett-Neto A. G. (2013). Seasonality and chemical elicitation of defense oleoresin production in field-grown slash pine under subtropical climate. Theor. Exp. Plant Physiol. 25, 56–61. 10.1590/S2197-00252013000100007

[B49] Rodrigues-Corrêa K. C. D. S. de Lima J. C. Fett-Neto A. G. (2012). Pine oleoresin: tapping green chemicals, biofuels, food protection, and carbon sequestration from multipurpose trees. Food Energy Secur. 1, 81–93. 10.1002/fes3.13

[B50] Rodrigues-Corrêa K. C. D. S. de Lima J. C. Fett-Neto A. G. (2013). Oleoresins from pine: production and industrial uses, in Natural Products, eds Ramawat K. G. Mérillon J.-M. (Berlin Heidelberg: Springer), 4037–4060.

[B51] Rodrigues-Corrêa K. C. S. Fett-Neto A. G. (2012). Physiological control of pine resin production, in Pine Resin: Biology, Chemistry and Applications, eds Rodrigues-Corrêa K. C. S. Fett-Neto A. G. (Kerala: Research Signpost), 25–48.

[B52] Savage T. J. Hatch M. W. Croteau R. (1994). Monoterpene synthases of *Pinus contorta* and related conifers. A new class of terpenoid cyclase. J. Biol. Chem. 269, 4012–4020. 8307957

[B53] Song J.-D. Kim J.-H. Lee D.-H. Rhew T. H. Cho S. H. Lee C.-H. (2005). Developmental regulation of the expression of 1-aminocyclopropane-1-carboxylic acid (ACC) synthase and ACC oxidase genes in hypocotyls of etiolated mung bean seedlings. Plant Sci. 168, 1149–1155. 10.1016/j.plantsci.2004.11.015

[B54] Stubbs J. Roberts D. R. Outcalt K. W. (1984). Chemical Stimulation of Lightwood in Southern Pines. Asheville: United States Department of Agriculture, General United States Department of Agriculture, Forest Service.

[B55] Trapp S. Croteau R. (2001). Defensive resin biosynthesis in conifers. Annu. Rev. Plant Physiol. Plant Mol. Biol. 52, 689–724. 10.1146/annurev.arplant.52.1.689 11337413

[B56] Tu L. Zhang X. Liu D. Jin S. Cao J. Zhu L. (2007). Suitable internal control genes for qRT-PCR normalization in cotton fiber development and somatic embryogenesis. Chinese Sci. Bull. 52, 3110–3117. 10.1007/s11434-007-0461-0

[B57] Vandesompele J. De Preter K. Pattyn F. Poppe B. Van Roy N. De Paepe A.. (2002). Accurate normalization of real-time quantitative RT-PCR data by geometric averaging of multiple internal control genes. Genome Biol. 3, research0034.1–research0034.11. 10.1186/gb-2002-3-7-research0034 12184808 PMC126239

[B58] Wang L. Xie W. Chen Y. Tang W. Yang J. Ye R.. (2010). A dynamic gene expression atlas covering the entire life cycle of rice. Plant J. 61, 752–766. 10.1111/j.1365-313X.2009.04100.x 20003165

[B59] Wang M. Wang Q. Zhang B. (2013). Evaluation and selection of reliable reference genes for gene expression under abiotic stress in cotton (*Gossypium hirsutum* L.). Gene 530, 44–50. 10.1016/j.gene.2013.07.084 23933278

