# Corrigendum: Reference Genes for qPCR Analysis in Resin-Tapped Adult Slash Pine As a Tool to Address the Molecular Basis of Commercial Resinosis

**DOI:** 10.3389/fpls.2018.00046

**Published:** 2018-01-23

**Authors:** Júlio C. de Lima, Fernanda de Costa, Thanise N. Füller, Kelly C. da Silva Rodrigues-Corrêa, Magnus R. Kerber, Mariano S. Lima, Janette P. Fett, Arthur G. Fett-Neto

**Affiliations:** ^1^Plant Physiology Laboratory, Center for Biotechnology and Department of Botany, Federal University of Rio Grande do Sul, Porto Alegre, Brazil; ^2^Biological Sciences Department, Regional Integrated University of Alto Uruguai and Missões (URI-FW), Frederico Westphalen, Brazil

**Keywords:** resin, Pinus, gene expression, normalizer genes, terpene synthase

In the original article, there was an error. We incorrectly described the concentration of the synthetic auxin 1-naphthaleneacetic acid (Treatment name: NAA). Rather than 10 mM, it should be corrected to 1.0 mM.

A correction has been made to: Materials and Methods, Plant Material and Resin Tapping Process, First Paragraph.

Approximately 16 years-old slash pine (*P. elliottii* Engelm. var. elliottii) trees with a diameter at breast height ranging from 65 to 90 cm, grown at Irani Celulose forest installations, Balneário Pinhal, RS, Brazil (30°14′S and 50°14′W), were used in the experiments. Resin tapping was performed according to a protocol previously established (Rodrigues et al., 2011) based on Pio and Valente (1998), using the “bark streak” system (Stubbs et al., 1984). Each stimulant paste contained 20% of sulfuric acid in aqueous solution and rice husk powder as an inert substrate to optimize paste consistency. Single modifications in the composition of the resin stimulant paste were done by including 1.0 mM of the synthetic auxin 1-naphthaleneacetic acid (Treatment name: NAA), 500 mM potassium sulfate (Treatment name: POTASSIUM) or 3% of 2-chloroethylphosphonic acid (Treatment name: CEPA) (v/v of active ingredient, an ethylene releasing agent). A negative control without paste application (bark streak only) was also included. The treatments and rationale for application are listed in **Table 1**.

The authors apologize for this error and state that this does not change the scientific conclusions of the article in any way.

The original article has been updated.

## Conflict of interest statement

The authors declare that the research was conducted in the absence of any commercial or financial relationships that could be construed as a potential conflict of interest.
