# Supplementary material for: Reference Genes for qPCR Analysis in Resin-Tapped Adult Slash Pine As a Tool to Address the Molecular Basis of Commercial Resinosis
Source: Front Plant Sci. 2016 Jun 16;7:849. doi: 10.3389/fpls.2016.00849 (PMC4909774; doi:10.3389/fpls.2016.00849)
Supplement: Table S1 — Description of target genes used to validate the putative reference genes. [file Table1.DOCX]

**Table S1. Description of target genes used to validate the putative reference genes**

| Gene | Gene abbreviation of putative genes | Function | Primer sequence  (5’-3’) (forward/reverse) | Amplicon length (bp) | Amplification efficiency | GenBank accession number |
| --- | --- | --- | --- | --- | --- | --- |
| **Pinus contorta (−)-β-pinene synthase** | *(-)βpinS1-*like | (-) β-pinene synthesis | ATGGATTTAATATCTGTCTTACCGTCGAC/  TTATAAAGGC ACAGGTTCAAGGAG | 148 | 1.842 | JQ240293 |
| **Pinus taeda (+)-α-pinene synthase** | *(+)αpinS-*like | (+)α-pinene synthesis | cgacaacatcaacgaaatgg/  tcgaacgtgggaagataacc | 147 | 1.834 | AAO61228 |
| **Pinus taeda α-farnesene synthase** | *αFS-*like | α-farnesene synthesis | TGGGAAGCTTTAATCGATGC/ GGAGAGTGGCTGCTCGATAC | 124 | 1.899 | AF543528.1 |
| **Pinus contorta Levopimaradiene/abietadiene synthase** | *LAS1-*like | abietadiene synthesis | GAATGCTCTGGAGGATACGG/ TCCAGCCTTGGCATACTTCT | 114 | 1.835 | JQ240310.1 |
