# Supplementary figures and images for: Reference Genes for qPCR Analysis in Resin-Tapped Adult Slash Pine As a Tool to Address the Molecular Basis of Commercial Resinosis
Source: Front Plant Sci. 2016 Jun 16;7:849. doi: 10.3389/fpls.2016.00849 (PMC4909774; doi:10.3389/fpls.2016.00849)

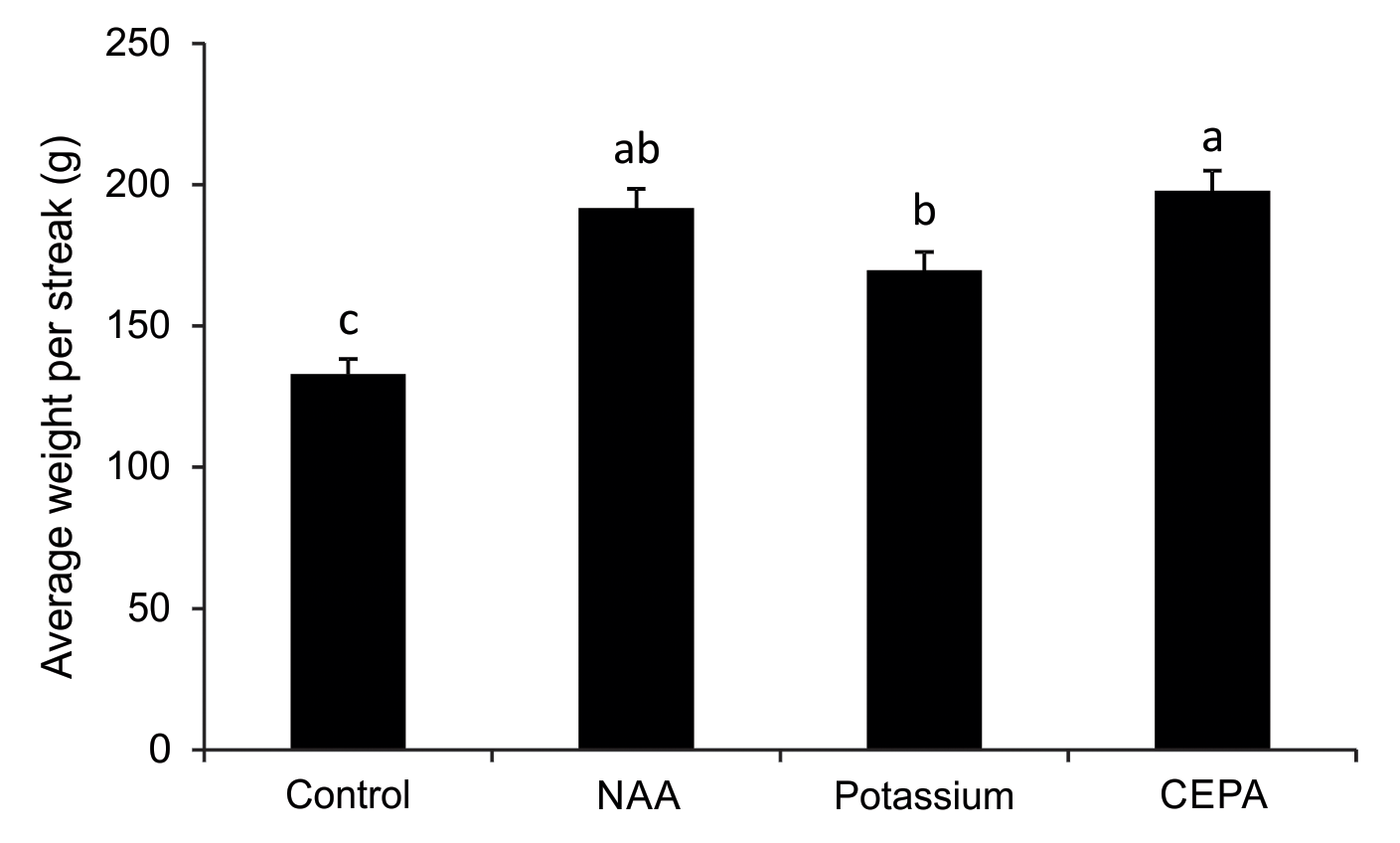

Supplement: Figure S1 — Resin biomass yield estimated as average per streak. Data for winter season based on 80 trees per stimulant paste treatment was used to estimate average resin yield per streak (corresponding to a 15 day period). Different letters indicate significant difference between treatments by Tukey test (P ≤ 0.05). [file Image1.TIF]

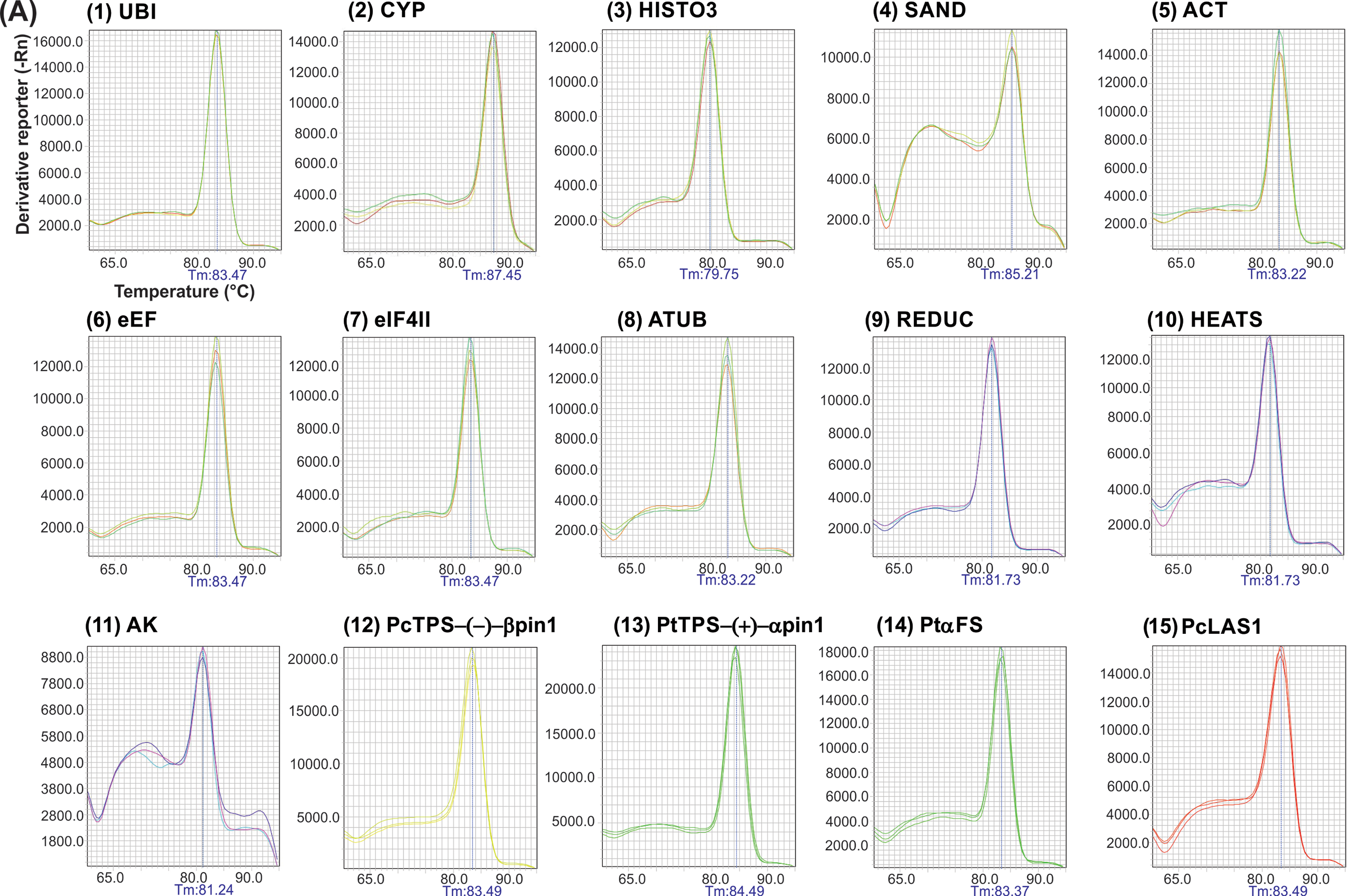

Supplement: Figure S2 — Specificity of qPCR. (A) Melting curves of the 11 reference genes plus target genes showing a single peak (each representing a technical triplicate). Y-axis: derivative reporter (-Rn) of the melting peak (random units); X-axis: temperature of melting; each vertical line marking the melting peak represents the medium temperature of melting between 80 and 90°C. (B) Agarose gel showing PCR amplicons of each gene. Numbers in the gel correspond to the ones in the melting curves. DNA ladder in the gel: 100 bp. [file Image2.TIF]

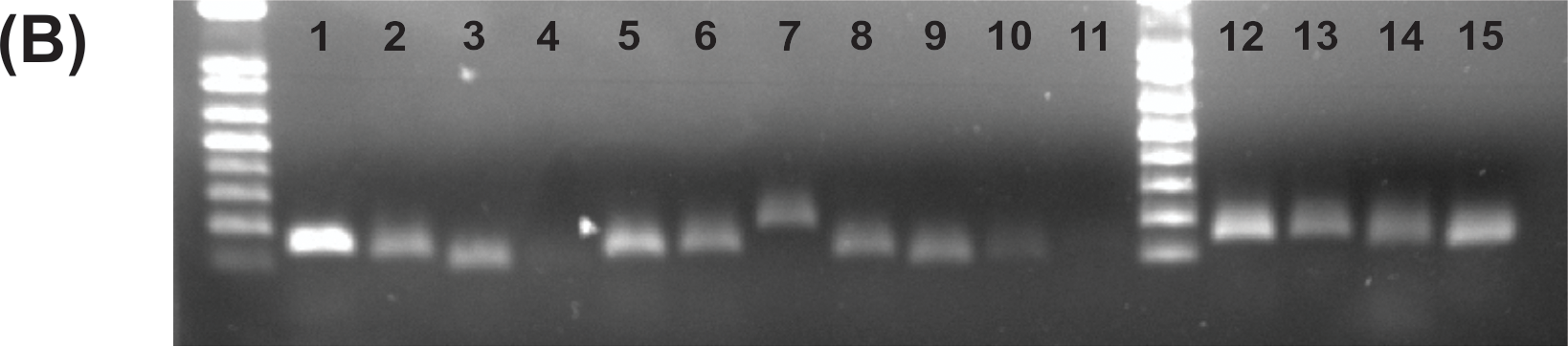

Supplement: Supplementary file 4 [file Image3.tif]
